# Clues from hypercalcaemia

**DOI:** 10.1038/sj.bjc.6600220

**Published:** 2002-04-08

**Authors:** S Nicholson, J Waxman

**Affiliations:** Department of Cancer Medicine, Faculty of Medicine, Imperial College of Science, Technology & Medicine, Hammersmith Campus, Du Cane Road, London W12 ONN, UK

## Abstract

*British Journal of Cancer* (2002) **86**, 1021–1022. DOI: 10.1038/sj/bjc/6600220 www.bjcancer.com

© 2002 Cancer Research UK

## 

The conundrum of hypercalcaemia in malignancy occurring in the absence of extensive bony metastases was finally solved in the late 1980s with the identification and cloning of PTHrP (parathyroid hormone-related protein) and the history of this discovery is summarised by Martin and Suva (1988). Original work in cell lines and a renal tumour was followed by the identification of PRHrP in a wide variety of malignances (Honda *et al*, 1988). PTHrP raises serum calcium and stimulates osteoclast activity, leading to bone destruction. This seemed to suggest a simple sequence of events, but malignancy, bony metastasis and hypercalcaemia are linked by mechanisms more subtle than any single linear chain of cause and effect. PTHrP shares eight of its first 13 amino acids with PTH (Mangin *et al*, 1988). This structural homology leads to shared receptor binding. There are biochemical differences in the effects of these two hormones. PTHrP is a more potent inhibitor of caliuria and promoter of phosphaturia than PTH. Circulating PTHrP is undetectable in health but present in around 80% of patients with humoral hypercalcaemia. Tissue expression of PTHrP is far more extensive than PTH, and this is reflected in a range of functions beyond calcium homeostasis (Strewler, 2000). The presence of PTHrP in the uterus and the stomach, coupled with its ability to relax smooth muscle, has led to the assertion that, in these tissues at least, it is PTHrP which is the true physiological effector molecule (Martin, 1996). The role of PTHrP in placental calcium transport seems, similarly, to imply far greater functional importance than that of PTH. It is the role of PTHrP in cartilage development, however, which may reveal its significance to the progression of malignant disease. When chondrocytes form new bone they first proliferate then produce the basic extracellular matrix which will be later invaded by cellular components of bone. The chondrocytes then apoptose. The absence of PTHrP, in PTHrP knock-out mice, leads to failure of proliferation and early apoptosis (Amizuka *et al*, 1996). This protection of chondrocytes from apoptosis in the physiological setting is mirrored in cancer, where transfection of PTHrP into cell lines confers resistance to apoptotic stimuli (Dougherty *et al*, 1999).

PTHrP stimulates osteoclastic bone resorption. Studies in breast cancer have shown that PTHrP is expressed in most breast primaries and bony metastases, but to a lesser extent in visceral metastases (Powell *et al*, 1991). The question then arises whether PTHrP is not merely a marker for bony metastases but also prognostic factor for progression. Animal experiments using a PTHrP-expressing cell line and blocking monoclonal antibody have demonstrated the importance of PTHrP in progression to metastatic bone disease (Guise *et al*, 1996), while cell culture work indicates that PTHrP can function as a transforming growth factor. There is, then, a high index of suspicion in breast cancer that PTHrP is a key factor in the development of bony metastases.

Prostate cancer has a different natural history to breast cancer. Hypercalcaemia is uncommon in prostate cancer and metastases are predominantly osteoblastic. It is important to realise, however, that the osteoblastic response seen in prostate cancer is preceded, at a cellular level, by osteoclast activation. The difference between breast and prostate, therefore, is not that osteoclast activation occurs or does not occur, but rather that in prostatic metastases an intense osteoblastic reaction is also present (Roodman, 2001). Elegant experiments using *in vivo* inocculation of paired transfected and untransfected cell lines have, furthermore, indicated a greater metastatic potential for the transfected lines (Rabbani *et al*, 1999) mirroring the results in breast cancer.

The paper by Bryden *et al*. (2002) in this issue of the journal extends observations of PTHrP expression in other tumours to prostate cancer. The authors have demonstrated a high rate of concordance for the expression of both PTHrP and its receptor in paired primary tumours and bony metastases. The ultimate purpose of studies such as these is either to identify new markers of prognostic importance, or to provide a mechanistic explanation of disease progression which may yield new therapeutic targets. The high frequency of expression in primary prostate cancers would seem to indicate that PTHrP is a poor discriminator of metastatic potential. However, the sample sizes are small, and larger studies linking PTHrP expression to outcome are required to demonstrate any prognostic significance of this observation. The role of experimental therapeutics directed against PTHrP may seem nonsensical, since the numerous factors which come into play by the time metastatic bone disease has developed implies that there is no role for PTHrP-directed intervention. If, however, a link between PTHrP and progression to bone disease is established, then targetting the PTHrP at earlier disease stages might eventually lead to a change in the natural history of prostate cancer.
